# Depth-Sensor-Based Monitoring of Therapeutic Exercises

**DOI:** 10.3390/s151025628

**Published:** 2015-10-09

**Authors:** Mu-Chun Su, Jhih-Jie Jhang, Yi-Zeng Hsieh, Shih-Ching Yeh, Shih-Chieh Lin, Shu-Fang Lee, Kai-Ping Tseng

**Affiliations:** 1 Department of Computes Science and Information Engineering, National Central University, Taoyuan City 32001, Taiwan; E-Mails: muchun@csie.ncu.edu.tw (M.-C.S.); fieldddog@gmail.com (J.-J.J.); yakumolin@gmail.com (S.-C.L.); 2 Department of Management and Information Technology, Southern Taiwan University of Science and Technology, Tainan City 71005, Taiwan; 3 School of Information Science and Technology, Fudan University, Shanghai 200433, China; E-Mail: yeshiqing@fudan.edu.cn; 4 Department of Rehabilitation, Landseed Hospital, Taoyuan City 324, Taiwan; E-Mails: leesf@landseed.com.tw (S.-F.L.), tsengkp@landseed.com.tw (K.-P.T.)

**Keywords:** SOM, motion trajectory, spatial-temporal pattern recognition, therapeutic exercise

## Abstract

In this paper, we propose a self-organizing feature map-based (SOM) monitoring system which is able to evaluate whether the physiotherapeutic exercise performed by a patient matches the corresponding assigned exercise. It allows patients to be able to perform their physiotherapeutic exercises on their own, but their progress during exercises can be monitored. The performance of the proposed the SOM-based monitoring system is tested on a database consisting of 12 different types of physiotherapeutic exercises. An average 98.8% correct rate was achieved.

## 1. Introduction

At the rehabilitation departments of hospitals in Taiwan, it is not unusual that several patients are assigned to one exercise physiologist or physical therapist during their rehabilitation treatment in a clinical room. One-on-one clinical service is not possible, therefore an exercise physiologist or a physical therapist cannot know how well his or her patients are practicing the assigned exercises. In addition, patients themselves usually do not have the enough knowledge about whether they are practicing physiotherapeutic exercises correctly, leading to ineffective rehabilitation and even producing adverse compensation effects. Therefore, to alleviate the burdens of exercise physiologists or physical therapists, a physiotherapeutic exercise monitoring system which can automatically measure how well a patient is practicing the suggested exercises deserves to be developed [1,2,3]. A good physiotherapeutic exercise monitoring system should be able to provide a measurement report about how well a patient is practicing a suggested exercise, facilitate the effective execution of the assigned rehabilitation programs, issue a warning alarm signal when an incorrect exercise is detected,* etc*. For this to happen, it requires an effective motion trajectory recognition algorithm which is able to evaluate whether the physiotherapeutic exercise performed by a patient matches the corresponding assigned exercise.

Automatic motion trajectory recognition turns out to be very challenging because motion trajectories are spatio-temporal patterns. Speech recognition and gesture recognition are another two typical examples of spatio-temporal pattern recognition problems. Several reviews of the topics related to the domain of human motion analysis can be found in [4,5,6,7,8,9,10]. Basically, there are three kinds of approaches to spatio-temporal pattern recognition. The simplest way is first to turn a temporal sequence into a spatial pattern and then to employ a template matching technique to compute the degree of similarity between the test pattern and the template pattern. The dynamic time warping (DTW) algorithm provides the effect of a non-linear normalization process in order to make the similarity measure operate successfully [11]. The DTW algorithm operates by stretching the template pattern and measuring the amount of stretching required. The less stretching needed, the more similar the patterns are. Although the DTW algorithm is easy to implement, it requires substantial computation to reach an optimal DTW path. The other approach is to train recurrent neural networks [12]. The time delay neural network (TDNN) whose hidden nodes and output nodes are replicated across time is one of the popular recurrent neural networks [13]. Time-delayed input frames allow the TDNN to be able to respond to time-varying signals, however, one may also find that it usually takes a lot of time to train a recurrent neural network. Another approach is to employ Hidden Markov Models (HMMs) to recognize spatial-temporal patterns. The basic idea behind the HMM is that a spatio-temporal signal can be characterized by a parametric stochastic process. A HMM can be thought of as a finite-state machine where the transitions between the states are governed by probabilistic laws. The key parameters to be determined in an HMM are: (1) the number of states in the model; (2) the state-transition probability distribution; (3) the observation symbol probability distribution; and (4) the initial-state distribution [14,15,16]. The price paid for HMM is that we have to collect a great amount of data and a lot of time is required to estimate the corresponding parameters in HMMs. In addition to those three popular approaches, recently, several different approaches to solve motion capture data have also been proposed [17,18,19,20,21,22,23,24,25,26,27]. For example, the concept of motion templates (MTs) which can capture the spatio-temporal characteristics of an entire motion class in a compact matrix representation was introduced in [17,19]. The idea of incorporating spatio-temporal invariance into the geometric features and adaptive segments was presented in [18]. Keogh* et al.* proposed a novel technique to speed up similarity search under uniform scaling based on bounding envelopes [20], and a novel fast method for global similarity searches was discussed in [21]. An efficient method for fully automatic temporal segmentation of human motion sequences was proposed in [22]. Baumann* et al.* presented a data-driven method for recognizing human full body actions from live motion data [23]. Morris* et al.* proposed REcoFit which is a system for automatically tracking repetitive exercises [24], while Beaudoin* et al.* presented a technique to automatically distill a motion-motif graph from an arbitrary collection of motion capture data [25], and Bernard* et al.* proposed an exploratory search and analysis system for sequences of human motion in large motion capture data collections [26]. Wilhelm* et al.* proposed a visual-interactive system for the exploration of horse motion data [27]. In the system, they employed the SOM algorithm as a non-linear projection technique to provide a data aggregation mechanism to facilitate visual overview.

Numerous reports have been written about the successful applications of the self-organizing feature map (SOM) algorithm [28]. These applications widely range from simulations used for the purpose of understanding and modeling of computational maps in the brain to subsystems for engineering applications [29]. Recently, several different approaches to the use of SOM in spatio-temporal pattern recognition have been proposed [30,31,32,33,34]. Gao* et al.* [30] and Fang* et al.* [31] used the SOM as an implicit feature extractor of different signers for continuous HMM. Corradini* et al.* regarded the SOM as a quantizer of 32 defined sub-gestures for discrete HMM [32]. Huang and Kuh proposed a neural network system which combines a SOM and a multi-layer perceptron (MLP) for the problem of isolated word speech recognition [33]. In one of our previous works [34], we proposed a SOMART system for the recognition of hand gestures. The proposed SOMART system integrates the SOM and the ART algorithm.

In this paper, we propose the SOM-based monitoring system of which core module is the SOM-based motion trajectory recognition algorithm. The algorithm starts with the generation of basic posture unit map. The sequence of a motion trajectory is transformed into a 2-D trajectory map based on the basic posture unit map. Then the problem of recognizing motion trajectories is transformed to the problem of recognizing 2-D trajectory maps. Finally, an unknown motion trajectory is classified to be the motion trajectory with the maximum similarity in the motion templates via a template matching technique.

The remainder of the paper is organized as follows: we first briefly review the SOM algorithm in Section 2. Then the proposed SOM-based motion trajectory recognition algorithm is discussed in Section 3. In Section 4, we give the results obtained by applying the algorithm to a database consisting of 12 different types of physiotherapeutic exercises. Finally, Section 5 concludes the paper.

## 2. Brief Review of the SOM Algorithm

The training algorithm proposed by Kohonen for forming a feature map can be summarized as follows [28]:

- Step 1: Initialization: Choose random values for the initial weights ${\underline{w}}_{j}(0)$.
- Step 2: Winner Finding: Find the winning neuron $j^{*}$ at time *k*, using the minimum-distance Euclidean criterion:
  (1) $$j^{*}=\arg\;\underset{j}{\min}\left\|\underline{x}(k)-{\underline{w}}_{j}\right\|,\;j=1,\cdots ,M\times N$$

where $\underline{x}(k)={\left[x_{1}(k),\cdots ,x_{n}(k)\right]}^{T}$ represents the *k*th input pattern, M×N is the total number of neurons, and ‖ ⋅ ‖ indicates the Euclidean norm. Depending on the application of interest, the response of the network could be either the index of the winning neuron or the weight vector [28].

- Step 3: Weight Updating: Adjust the weights of the winner and its neighbors, using the following rule:

(2) $${\underline{w}}_{j}(k+1)={\underline{w}}_{j}(k)+\eta (k){\text{Λ}}_{j^{*}}(k)[\underline{x}(k)-{\underline{w}}_{j}(k)]$$

where $\eta (k)=\eta_{0}\left(1-\frac{k}{{\text{N}}_{\text{K}}}\right)$, N_K_ is the number of iterations, ${\text{Λ}}_{j^{*}}(k)$ is the topological neighborhood function of the winner neuron $j^{*}$ at time *k*. A typical choice of ${\text{Λ}}_{j^{*}}(k)$ is the Gaussian-type function:

(3) $${\text{Λ}}_{j^{*}}(k)=\exp(-\frac{d_{j^{*},j}^{2}}{2\sigma {\left(k\right)}^{2}})$$

where the parameter σ(k) is the “effect width” of the topological neighborhood and $d_{j^{*},j}^{2}$ is the lateral distance between neurons $j^{*}$ and j in the discrete output space. The “effect width” measures the degree to which the excited neurons in the vicinity of the winning neuron [28]. It should be emphasized that the success of the map formation is critically dependent on how the values of the main parameters (*i.e.*, η(k) and ${\text{Λ}}_{j^{*}}(k)$), initial values of weight vectors, and the number of iterations are predetermined. In our experiments discussed in Section 4, the initialization method proposed in [35] was utilized to initialize the weight vectors to quickly construct a good initial map. As for the values of the main parameters (*i.e.*, η(k) and ${\text{Λ}}_{j^{*}}(k)$), we set $\eta_{0}=0.1$, ${\text{N}}_{\text{K}}=100$, and σ(k)=1.0. Depending on the application of interest, the response of the network could be either the index of the winning neuron or the weight vector [17].

- Step 4: Continuation. Increment time step k by one and go back to step 2 until some kind of termination criterion is met.

## 3. The SOM-Based Motion Trajectory Recognition Algorithm

**Figure 1:**
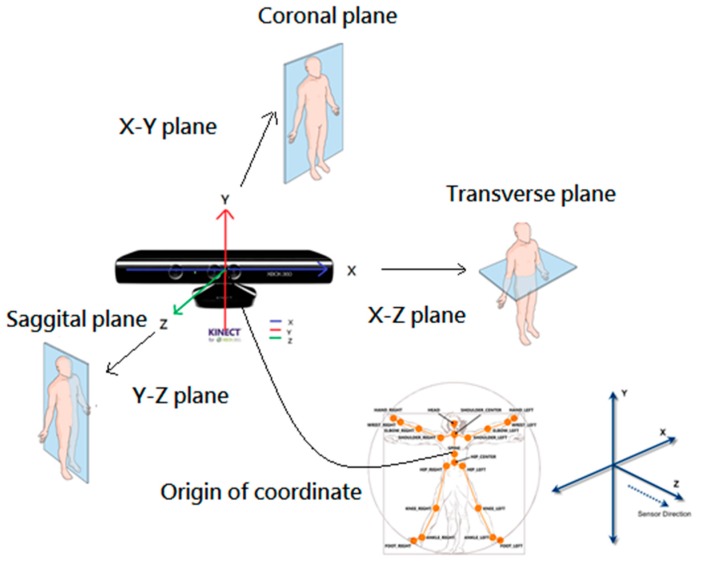
The body-plane coordinates transformation.

Before we explain how the proposed SOM-based motion trajectory recognition algorithm works, we need to first introduce how we capture the information about the motion trajectories. The motion trajectories are captured via a Kinect sensor. To make the features insensitive to personal height, the standing distance from the user to the Kinect sensor, and the facing orientation with respect to the Kinect sensor, we need to adopt an effective coordinate transformation. In this paper, we propose the body-plane coordinate transformation to transform the original world coordinates to the new coordinate system with the origin of coordinate centered at the spine location of the user. Then the X-Y plane is transformed to the coronal plane, the Y-Z plane to the sagittal plane, and the X-Z plane to the transverse plane, as shown in Figure 1.

**Figure 2:**
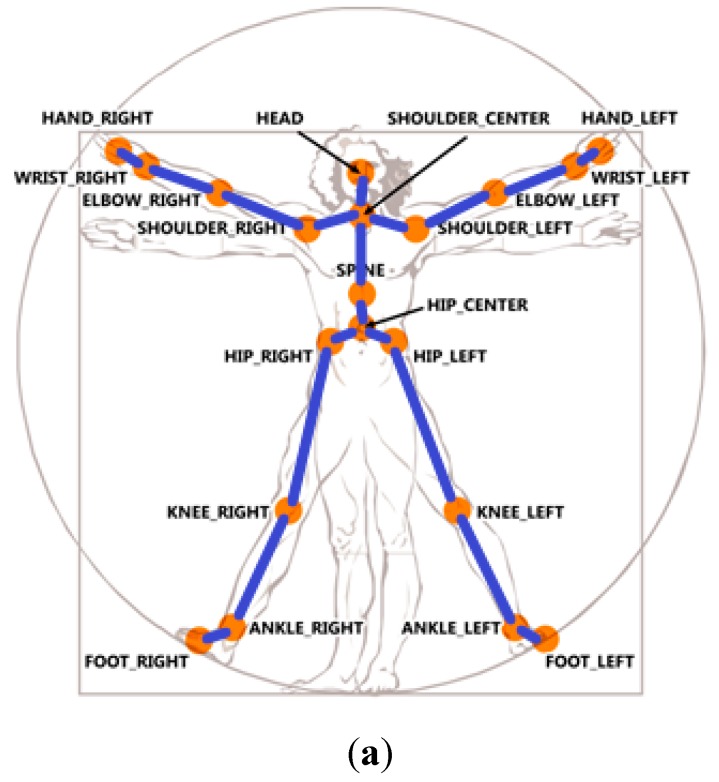

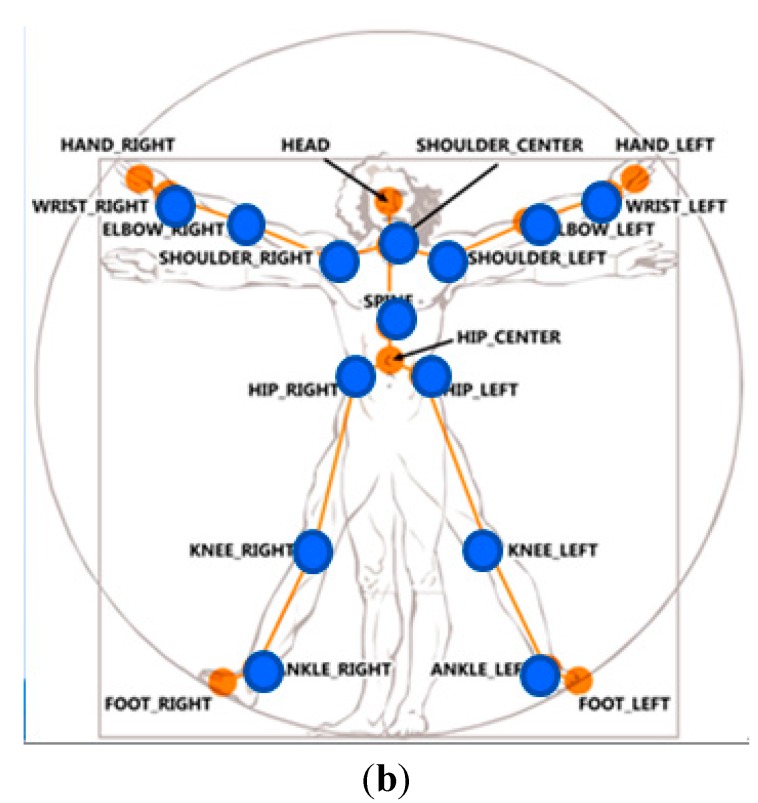
The 71 features for the motion trajectory extracted from the skeleton information provided from a Kinect sensor. (**a**) The 19 unit vectors; (**b**) The 14 joints.

To implement the body-plane transformation, we need three orthogonal vectors to form the new coordinates located at the spine position of the user. These three orthogonal vectors consist of the vector from the pine position to the shoulder center position, the vector from the pine position to the shoulder left position, and the cross product vector of the former two vectors. Based on the skeleton information provided from the Kinect sensor, we decide to choose 19 unit vectors of the body segment vectors as shown in Figure 2a and 14 joint angles as shown in Figure 2b as the features for each image frame of a motion trajectory. The joint angle is the angle between two connected body segments. Therefore, the feature vector is a 71-dimendional vector because the total dimensions for the 19 unit vectors are 3 × 19 and the total dimensions for the 14 joint angles are 14.

The proposed SOM-based motion trajectory recognition algorithm involves the following four steps:

Step 1. Generating a basic posture unit map

We use a data set consisting of many different motion trajectories practiced by different persons to run the SOM algorithm to construct a topologically preserved map with *M* × *N* neurons. The main mission of the SOM algorithm is to cluster motion trajectories into *M* × *N* small clusters. In our simulations, we found that the size 10 × 10 could produce a good recognition result. Basically, these 100 clusters can directly be used to serve as the 100 basic posture units for motion trajectories. Based on the 100 basic posture units, an individual motion trajectory can be represented as a sequence of a combination of 100 different basic postures with the sequence length equal to the number of the frames of the trajectory. To increase the tolerance to the person variability problem, we do not directly make each neuron to correspond to a basic posture unit. We fully utilize the topology-preserving characteristic of the SOM algorithm and then merge neighboring small clusters of the SOM map to form so-called “superclusters”. This merging process can reduce the number of basic posture units from 100 to the number of “superclusters”. Each supercluster then contains many small clusters. Via the use of superclusters, we allow each basic posture unit to have many templates so the sensitivity of the person variability can be reduced to some extent. We first use the U-matrix algorithm [36,37] to transform the trained map with 10 × 10 neurons into a digital image with 10 × 10 pixels. In the U-matrix algorithm, distances of each neuron to each of its immediate neighbors are calculated and visualized using gray shade. High values on the U-matrix mean large distance between neighboring map units, and thus indicate cluster borders. Clusters are typically uniform areas of low values and the number of clusters has to be determined by visualization. To more objectively determine the number of clusters, the watershed algorithm [38] is then adopted to segment the U-matrix image into several catchment basins. A simple merging scheme is adopted to merge shallow basins to their neighboring deep basins in order to prevent over-segmentation. The posture located at a basin is the most representative posture for the neurons located at the corresponding basin.

**Figure 3:**
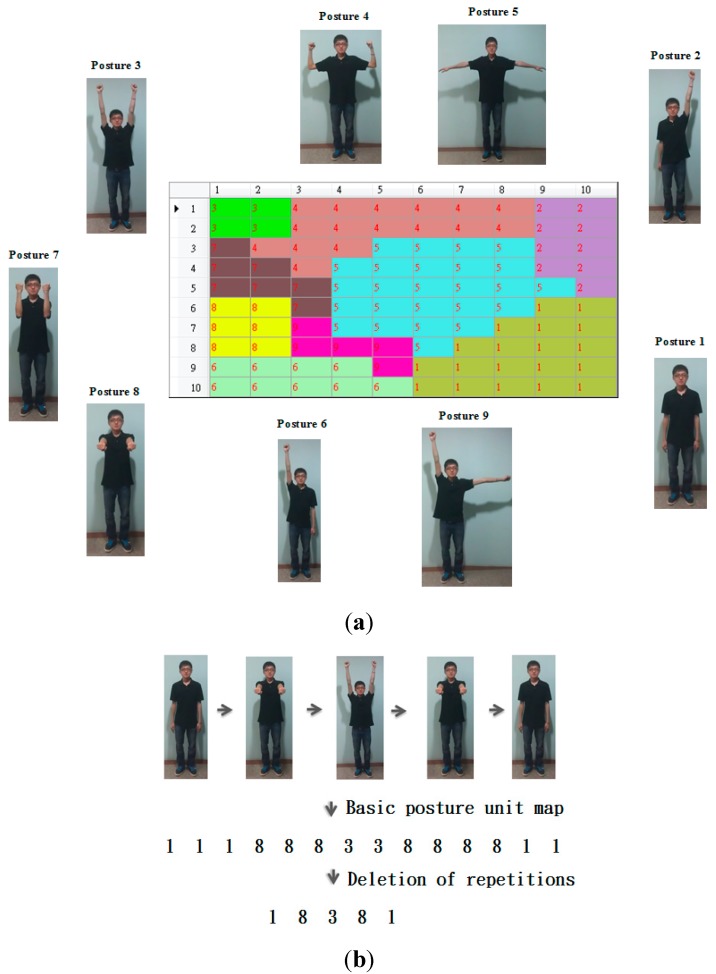
The basic posture unit map. (**a**) The basic posture map consisted of nine basic postures (**b**) A motion trajectory and its corresponding sequence.

The nine resultant basic postures are shown in Figure 3a. For example, while the first basic posture represents the postures located at the lower right corners, the second basic posture unit represents the postures located at the upper right corners. By viewing Figure 3a, we may find that these two basic postures are similar except that the left hand points up at the second basic posture. This observation matches the topological characteristic of the SOM algorithm. Based on the basic posture map, each motion trajectory can be transformed into a sequence of basic postures. For example, a motion trajectory shown in Figure 3b can be represented as a sequence, 11188833888811. Since repetitions contain no further information, we will delete repetitions in the sequence and then use the sequence 18,381 to represent the motion trajectory.

Step 2. Transforming a motion trajectory into a trajectory map

**Figure 4:**
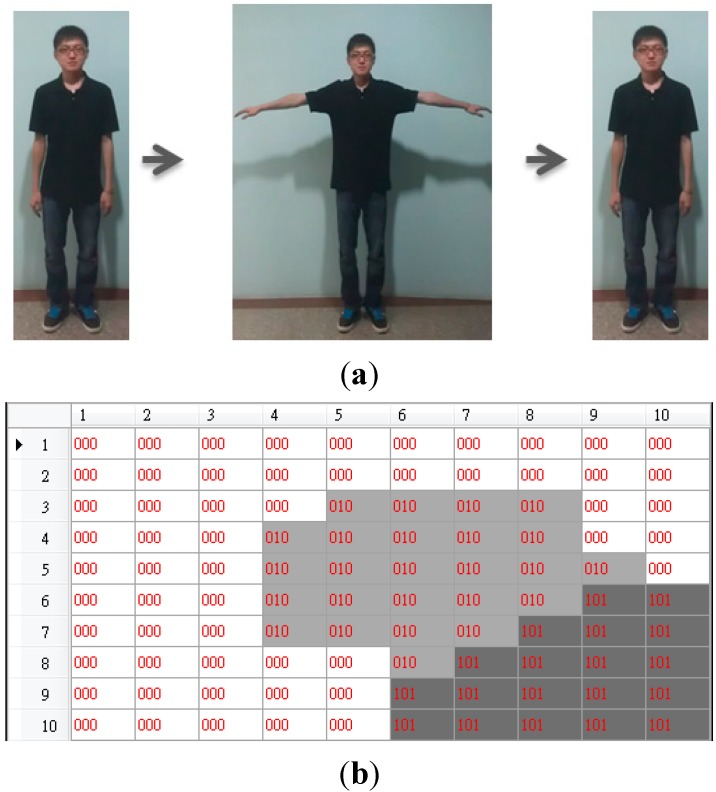
The trajectory map. (**a**) The motion trajectory and its corresponding sequence 151; (**b**) The resultant trajectory map.

As mentioned in the previous step, each motion trajectory can be represented as a sequence of nine symbols (*i.e.*, nine basic postures). We do not directly use a one-dimensional sequence to represent a motion trajectory because the length will vary with different motion trajectories. Therefore, we propose the use of a 2-dimensional trajectory map with a fixed size to represent a motion trajectory. The resultant sequence is projected on the basic posture map to generate a so-called “trajectory map” in the following way. Each frame of a motion trajectory is sequentially input to the basic posture map to locate the corresponding winner on the map. The basin corresponding to the winner will be rendered using a gray shade. Darker shades on the trajectory map mean earlier frames. Therefore, a motion trajectory will result in a trajectory map. Via this kind of rendering scheme, the temporal information will be retained in the trajectory map. We use an example shown in Figure 4 to illustrate how to generate a trajectory map. The motion trajectory shown in Figure 4a is consisted of basic posture units 1 and 5. Its corresponding sequence is 151. Therefore, its corresponding trajectory map is shown in Figure 4b where the gray levels for the basic posture units, 2, 3, 4, 6, 7, 8, and 9 are all white except the two regions corresponding to the basic postures 1 and 5. The binary string labeled at each cell of the basic posture unit map shown in Figure 4b represents the appearance orders of the corresponding basic postures. For example, the corresponding sequence is 151; therefore, there are three digits in the binary string. The first basic posture appears twice at the sequence and appears at the first time and the third time respectively; therefore, the binary string 101 is labeled at the region of the first basic posture located at the right bottom corner of the basic posture map.

Step 3. Generating trajectory map templates

Human variability causes the same type of a motion trajectory to be conducted differently each time. Even for the same person, different instances of a motion trajectory may not be identical. Therefore, a motion trajectory may result in more than one trajectory maps. There are two approaches to solve the variability problem. One approach is to store multiple templates for each motion trajectory. A problem associated with this kind of approach is the determination of the number of templates. The other approach is to generate one most representative template for each motion trajectory. We adopt the second approach to generate one template for each motion trajectory. We collect many instances for each motion trajectory and then the longest common subsequence (LCS) algorithm is adopted to search the longest common subsequence of multiple sequences [39]. An example is given in Table 1. In this example, there were three kinds of motion trajectories and four different users were asked to execute the motion trajectory for one time. Each motion trajectory is first converted into a sequence of basic postures via the basic posture unit map. Then resultant template for each kind of trajectory is shown at the last row of the table.

**Table 1:** The example of finding the template for each motion trajectory.

User	Trajectory 1	Trajectory 2	Trajectory 3
**1**	6, 1, 2, 1, 6	6, 11, 9, 11, 6	6, 1, 3, 6
**2**	6, 1, 2, 9, 2, 1, 3, 6	6, 10, 9, 10, 11, 6	6, 1, 5, 3, 6
**3**	6, 2, 1, 6	6, 11, 10, 9, 6, 11,6	6, 5, 1, 5, 3, 6
**4**	6, 5, 4, 2, 1, 6	6, 10, 9, 11, 6	6, 1, 6
**LCS**	6, 2, 1, 6	6, 10, 9, 11, 6	6, 1, 6

Step 4. Decision making

To classify an unknown motion trajectory, we first project the sequence of the motion trajectory into the basic posture unit map to create the corresponding trajectory map. Then we match the trajectory map with the templates of the motion trajectories in the database. Finally, the unknown motion trajectory is classified to be the motion trajectory with the largest similarity in the database. One immediate problem is how to measure the similarity degree between two trajectory maps. The simplest way to measure the similarity degree between two trajectory maps is the use of the image correlation computation. The similarity degree* S_k_* between the present trajectory map to be recognized, *p*(*x*, *y*), and the template map for the *k*th motion trajectory, *t_k_* (*x*, *y*), can be computed as follows:

(4) $$S_{k}=\frac{\sum_{x}\sum_{y}[p(x,y)-\bar{p}(x,y)][t_{k}(x,y)-\bar{t_{k}}(x,y)]}{{\left\{\sum_{x}\sum_{y}{\left[p(x,y)-\bar{p}(x,y)\right]}^{2}\sum_{x}\sum_{y}{\left[t_{k}(x,y)-\bar{t_{k}}(x,y)\right]}^{2}\right\}}^{½}}$$

where $\bar{p}\left(x,y\right)$ and $\bar{t_{k}}\left(x,y\right)$ represent the average value of *p*(*x*, *y*) and *t_k_* (*x*, *y*), respectively. If the computed similarity degree is below a pre-specified threshold, then the test motion trajectory is claimed to be not similar to any template in the database.

## 4. Experimental Results

**Figure 5:**
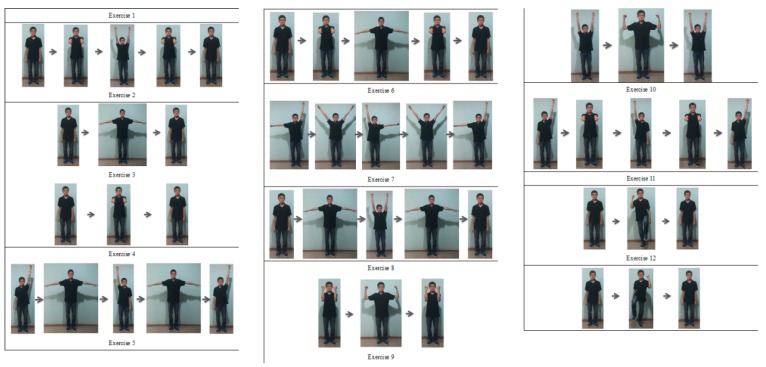
The 12 exercises recommended for patients with Parkinson’s disease.

To evaluate the performance of the proposed SOM-based motion trajectory recognition algorithm, four databases consisting of 12 physiotherapeutic exercises recommended for patients with Parkinson’s disease were generated. These 12 physiotherapeutic exercises are shown in Figure 5. Ten subjects as shown in Table 2 were invited to generate the four databases shown in Table 3.

**Table 2:** The ten persons invited to generate the four databases.

Subject	Gender	Height (cm)	Weight (kg)
1	Male	176	58
2	Female	162	47
3	Male	174	68
4	Male	177	72
5	Male	184	78
6	Female	160	43
7	Male	173	64
8	Female	163	45
9	Male	173	68
10	Male	170	65

**Figure 6:**
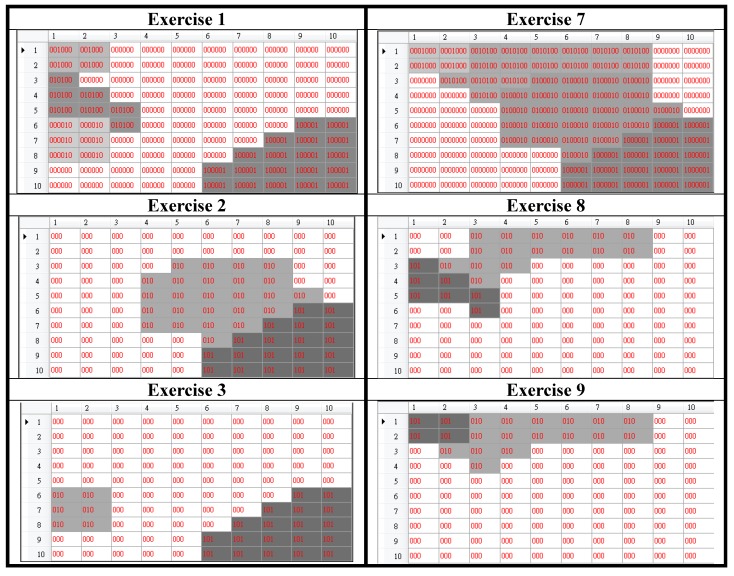

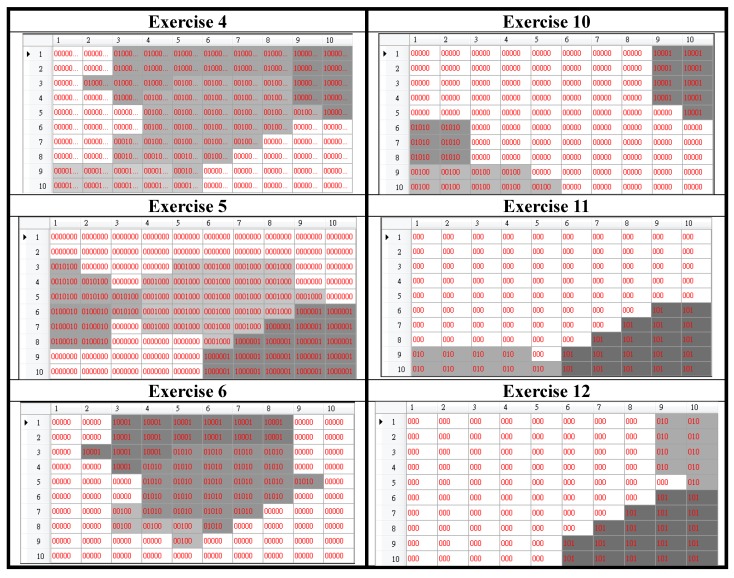
The 12 motion trajectory maps generated from the first database.

The first five subjects including four males and one female were asked to perform the 12 exercises. Each subject practiced each exercise for one time to generate the first database called the template database. This database was used to generate the basic posture unit map as shown in Figure 3a. Then we used the LCS method to generate one template for each exercise from the trajectories practiced by five persons. The resultant motion trajectory maps were shown in Figure 6. Obviously, these 12 trajectory maps look different. This observation confirms that the idea about representing motion trajectories in terms of trajectory maps works. These 12 trajectory maps were then stored as the templates for the use of testing the following four experiments.

**Table 3:** The four databases generated for testing the performance of the proposed method.

Database	Name	Subjects	Exercises
1	The template database	S1, S2, S3, S4, S5	Each exercise for one time
2	The user dependent database	S1, S2, S3, S4, S5	Each exercise for ten times
3	The user robustness database	S1, S2, S3, S4, S5	Each exercise for ten times under three different conditions (*i.e.*, exercise speed, pause, and facing orientation)
4	The user independent database	S6, S7, S8, S9, S10	Each exercise for ten times

Experiment One: User Dependent Test

We asked the same five subjects to practice the 12 exercises again. Each exercise was repeated ten times for each person to generate the second database called the user dependent database. The user dependent database was used for the purpose of testing. The experimental results showed that the average recognition rates achieved by the proposed method were 100% correct as shown in Table 4. For the comparison purpose, we adopted the popular DTW method and Support Vector Machines (SVMs) to test the second database. The unknown motion trajectory is classified to be the motion trajectory with the smallest accumulated Euclidean distance in the database. Since there were five trajectories for each exercise, one randomly chosen trajectory was used for the template and the remaining four trajectories were used for testing. The recognition performance achieved by the DTW method was 78.3% correct. As for the training of the SVMs, we used the template database to train SVMs (*i.e.*, each exercise has only one training data point). The inputs presented to the SVMs were the trajectory map. Then the user dependent database was used to test the trained SVMs. The recognition performance achieved by the SVMs was 83.0% and 91.7% correct with the standard Gaussian radial basis function kernels and the polynomial kernels, respectively. Obviously, our proposed method outperformed the DTW method and the SVMs based on the comparison of correct recognition rate.

**Table 4:** The recognition performance achieved by the proposed method.

	Subject	1	2	3	4	5	Average
Type							
**1**		100%	100%	100%	100%	100%	100%
**2**		100%	100%	100%	100%	100%	100%
**3**		100%	100%	100%	100%	100%	100%
**4**		100%	100%	100%	100%	100%	100%
**5**		100%	100%	100%	100%	100%	100%
**6**		100%	100%	100%	100%	100%	100%
**7**		100%	100%	100%	100%	100%	100%
**8**		100%	100%	100%	100%	100%	100%
**9**		100%	100%	100%	100%	100%	100%
**10**		100%	100%	100%	100%	100%	100%
**11**		100%	100%	100%	100%	100%	100%
**12**		100%	100%	100%	100%	100%	100%
**Average**		100%	100%	100%	100%	100%	100%

Experiment Two: Robustness Test

To test the robustness (e.g., exercise speed, pause, and facing orientation) of the proposed method, the third database called the robustness database was generated. The same five subjects were asked to perform the 12 exercises under three different conditions for ten times. The first condition was that the exercise speed was about two times slower than the speed they generated the second database. The second condition was that each person intended to pause for a little while during each exercise practice. The third condition was that they rotated their bodies with respect to the Kinect sensor about 45°. The performance was tabulated in Table 5. The average recognition rate was 99.20% correct. It means that the proposed method was very robust since the recognition rate just degraded for 0.8%. Based on these observations, our proposed algorithm seems promising. One thing should be emphasized is that if the facing orientation with respect to the Kinect sensor is larger than 45°, then the recognition performance will greatly degrade since the Kinect sensor is unable to stably capture the skeletal information.

**Table 5:** The robustness performance achieved by the proposed method.

	Subject	1	2	3	4	5	Average
Type							
**1**	Slow speed	100%	100%	100%	100%	100%	100%
	Pause	100%	100%	100%	100%	100%	100%
	45°	100%	100%	100%	100%	100%	100%
**2**	Slow speed	100%	100%	100%	100%	100%	100%
	Pause	100%	100%	100%	100%	100%	100%
	45°	100%	100%	100%	100%	100%	100%
**3**	Slow speed	100%	100%	100%	100%	100%	100%
	Pause	100%	100%	100%	100%	100%	100%
	45°	100%	100%	100%	100%	100%	100%
**4**	Slow speed	100%	100%	100%	100%	100%	100%
	Pause	100%	100%	100%	100%	100%	100%
	45°	100%	100%	100%	100%	100%	100%
**5**	Slow speed	100%	100%	100%	100%	100%	100%
	Pause	100%	100%	100%	100%	100%	100%
	45°	100%	100%	100%	100%	100%	100%
**6**	Slow speed	100%	90%	100%	90%	100%	96%
	Pause	100%	100%	90%	100%	100%	98%
	45°	100%	100%	90%	90%	90%	94%
**7**	Slow speed	100%	100%	100%	100%	100%	100%
	Pause	100%	100%	100%	100%	100%	100%
	45°	100%	100%	100%	100%	100%	100%
**8**	Slow speed	100%	100%	100%	100%	100%	100%
	Pause	100%	100%	100%	100%	100%	100%
	45°	100%	100%	100%	100%	100%	100%
**9**	Slow speed	100%	90%	90%	100%	100%	96%
	Pause	100%	100%	100%	90%	90%	96%
	45°	90%	90%	100%	90%	100%	94%
**10**	Slow speed	100%	100%	100%	100%	100%	100%
	Pause	100%	100%	100%	100%	100%	100%
	45°	100%	100%	100%	100%	100%	100%
**11**	Slow speed	100%	100%	100%	100%	100%	100%
	Pause	100%	100%	100%	100%	100%	100%
	45°	100%	100%	100%	100%	100%	100%
**12**	Slow speed	100%	100%	100%	100%	100%	100%
	Pause	100%	100%	100%	100%	100%	100%
	45°	100%	100%	100%	100%	100%	100%
**Average**		100%	99%	99%	99%	99%	99.2%

Experiment Three: User Independent Test

To test whether the proposed recognition algorithm is user-independent, the remaining five subjects including three males and two females were asked to perform the 12 exercises. Each exercise was repeated for ten times for each subject to generate the fourth database called the user independent database. The fourth database was used for testing purpose. The experimental results showed that the average recognition rates achieved by the proposed method were 97.7% correct as shown in Table 6. Although the recognition performance degraded from 100% to 97.7%, the average recognition rate was still high. It means that the proposed recognition algorithm had good generation ability.

**Table 6:** The recognition performance achieved by the proposed method for the user-independent test.

	Subject	6	7	8	9	10	Average
Type							
**1**		100%	100%	100%	100%	100%	100%
**2**		100%	100%	100%	100%	100%	100%
**3**		100%	100%	100%	100%	100%	100%
**4**		100%	100%	100%	100%	100%	100%
**5**		100%	100%	100%	100%	100%	100%
**6**		70%	90%	100%	100%	70%	86%
**7**		100%	100%	100%	100%	100%	100%
**8**		100%	100%	100%	100%	100%	100%
**9**		90%	100%	90%	90%	60%	86%
**10**		100%	100%	100%	100%	100%	100%
**11**		100%	100%	100%	100%	100%	100%
**12**		100%	100%	100%	100%	100%	100%
**Average**		96.7%	99.2%	99.2%	99.2%	94.2%	97.7%

Experiment Four: Grid Size Influence Test

**Figure 7:**
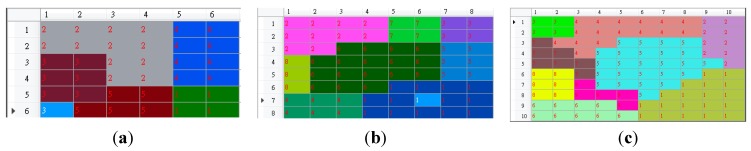
The basic posture unit maps resulted from grid size: (**a**) 6 × 6, (**b**) 8 × 8, and (**c**) 10 × 10.

In this experiment, we wanted to determine whether the grid size influences the recognition performance. Three different grid sizes, 6 × 6, 8 × 8, and 10 × 10 were compared here. The basic posture unit maps resulting from these three different grid sizes are shown in Figure 7.

**Figure 8:**
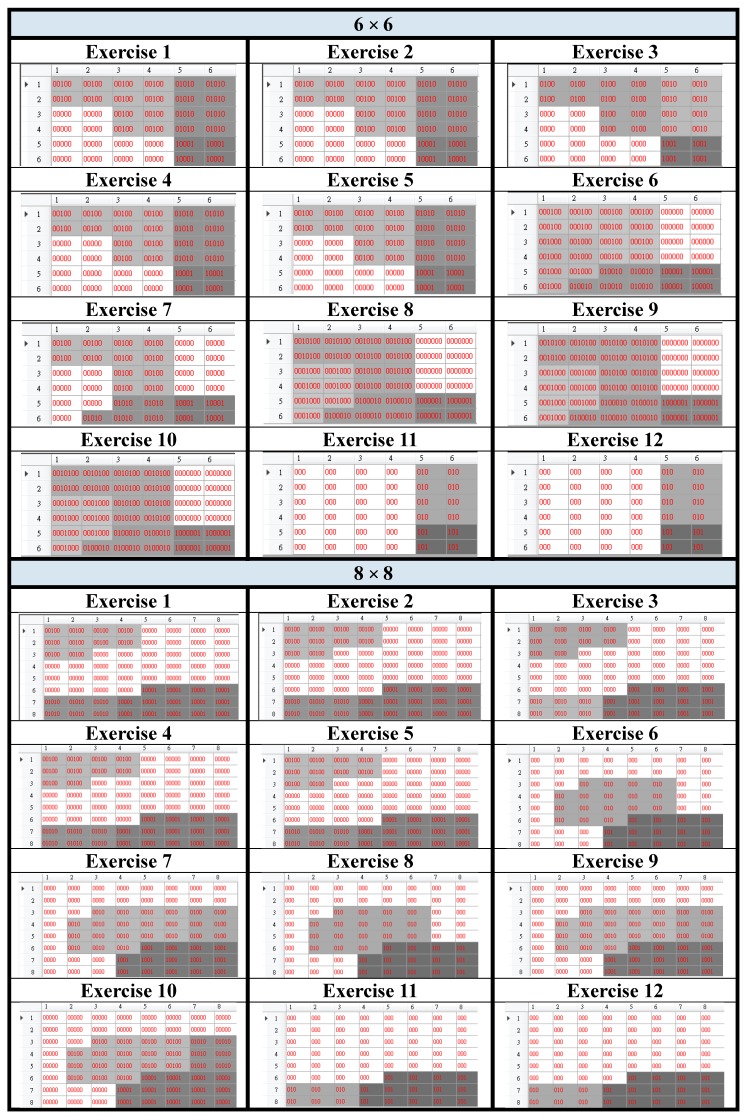

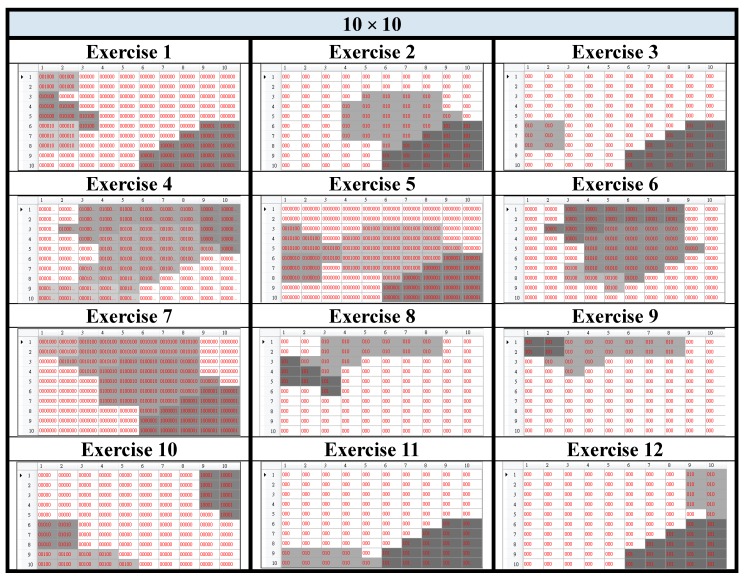
The trajectory maps resulted from grid size: 6 × 6, 8 × 8, and 10 × 10.

The numbers of basic posture units were 5, 8, and 9, for 6 × 6, 8 × 8, and 10 × 10, respectively. The three resultant trajectory maps were shown in Figure 8. Then we used the user dependent database to test the recognition performance of the three different grid sizes. The performance comparison was tabulated in Table 7. Several interesting observations could be concluded as follows. First of all, the larger the grid size, the larger the number of the basic posture units. Secondly, the larger the grid size, the better the recognition performance. Thirdly, there existed more difference among the trajectory maps as the grid size increases; therefore, the recognition performance was improved as the grid size increased. Based on these observations, if we want to recognize more motion trajectories then we need a larger grid size to exhibit enough differences among trajectory maps.

**Table 7:** The recognition performance achieved by the three different grid sizes.

**Grid Size**	6 × 6	8 × 8	10 × 10
**Performance**	33.67%	67%	100%

## 5. Conclusions

A method of recognizing spatio-temporal motion trajectories was proposed in this paper. Based on the proposed SOM-based motion trajectory recognition algorithm, we are able to implement a physiotherapeutic exercise monitoring system which can automatically measure how well a patient is practicing the suggested exercises. We used three databases to evaluate the effectiveness of the proposed SOM-based motion trajectory recognition algorithm. The average recognition rate that could be achieved was at least 97.7%. The robustness of the proposed algorithm was also verified by the second experiment.

Although the SOM algorithm has been applied in motion capture data for several years now, the major contribution of our method is the use of trajectory maps. Via a special kind of the proposed rendering scheme, the temporal information can be retained in the trajectory map. In addition, we adopt the image correlation computation to measure the similarity degree between two trajectory maps.

Since the Kinect depth sensor is more or less subject to the deployment environment (e.g., the distance between the sensor and the user, orientations, the lighting,* etc.*), our method is suitable for coarse-grained movements such as the twelve exercises shown in Figure 5. As for some more complex or subtle rehabilitation exercises such as palmar pinch, wrist motion (e.g., flexion, extension, and deviation), hand-axis motion (e.g., pronation and supination),* etc.*, our present method may not work well. This can be partly attributed to the features of the Kinect sensor because the current Kinect sensor is inadequate to provide the fine-grained rotation information about the fingers and the wrists. Inexpensive tracking cameras can provide enough information about the fine-grained rotation exercises, so we will try to generalize our method to those complex or subtle rehabilitation exercises to meet the needs of physiotherapy patients in the future.
